# Supplementary material for: Transposable elements maintain genome-wide heterozygosity in inbred populations
Source: Nat Commun. 2022 Nov 17;13:7022. doi: 10.1038/s41467-022-34795-4 (PMC9672359; doi:10.1038/s41467-022-34795-4)
Supplement: Supplementary file 1 — Supplementary Information [file 41467_2022_34795_MOESM1_ESM.pdf]

Transposable elements maintain genome-wide heterozygosity in inbred populations

De Kort *et al.*

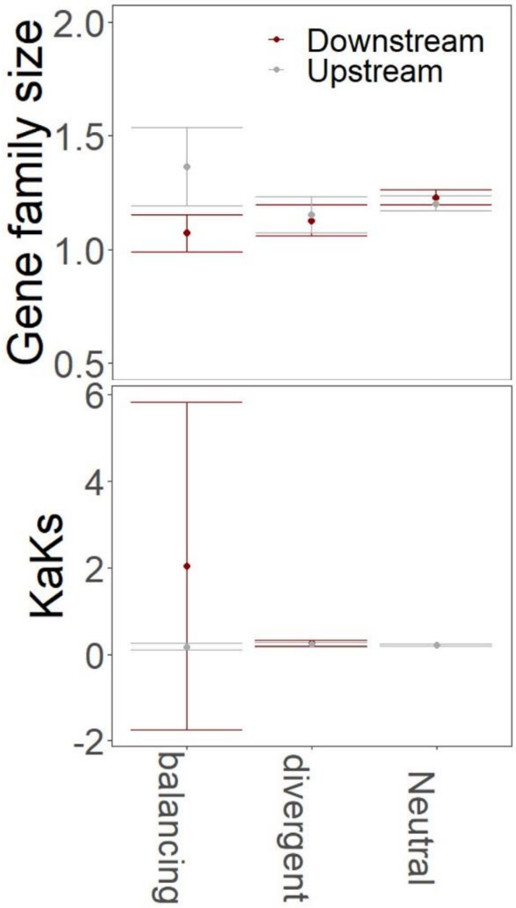


```
Analysis of Variance Table

Response: GeneFamilies
                   Df Sum Sq Mean Sq F value  Pr(>F)
Selection           2   3.20 1.59840  3.6012 0.02752 *
Sense               1   0.17 0.17401  0.3920 0.53133
Selection:Sense     2   2.32 1.16138  2.6166 0.07338 .
Residuals        1545 685.76 0.44386
```

```
Analysis of Variance Table

Response: KaKs
                 Df  Sum Sq Mean Sq F value    Pr(>F)
Selection         2   43.40 21.7017  8.2836 0.0002718 ***
Sense             1    2.45  2.4526  0.9362 0.3335194
Selection:Sense   2   41.88 20.9388  7.9924 0.0003618 ***
Residuals       929 2433.83  2.6198
```

Supplementary Figure 1. Gene essentiality variation across signatures of selection. Genes with SNPs under balancing selection have a higher proportion of single gene copies (reduced gene family size) and an increased proportion of non-synonymous SNPs (Ka) when downstream of transposable elements, pointing to essential genes downstream of TEs being particular targets of balancing selection. Data are presented as mean values +/- SEM.

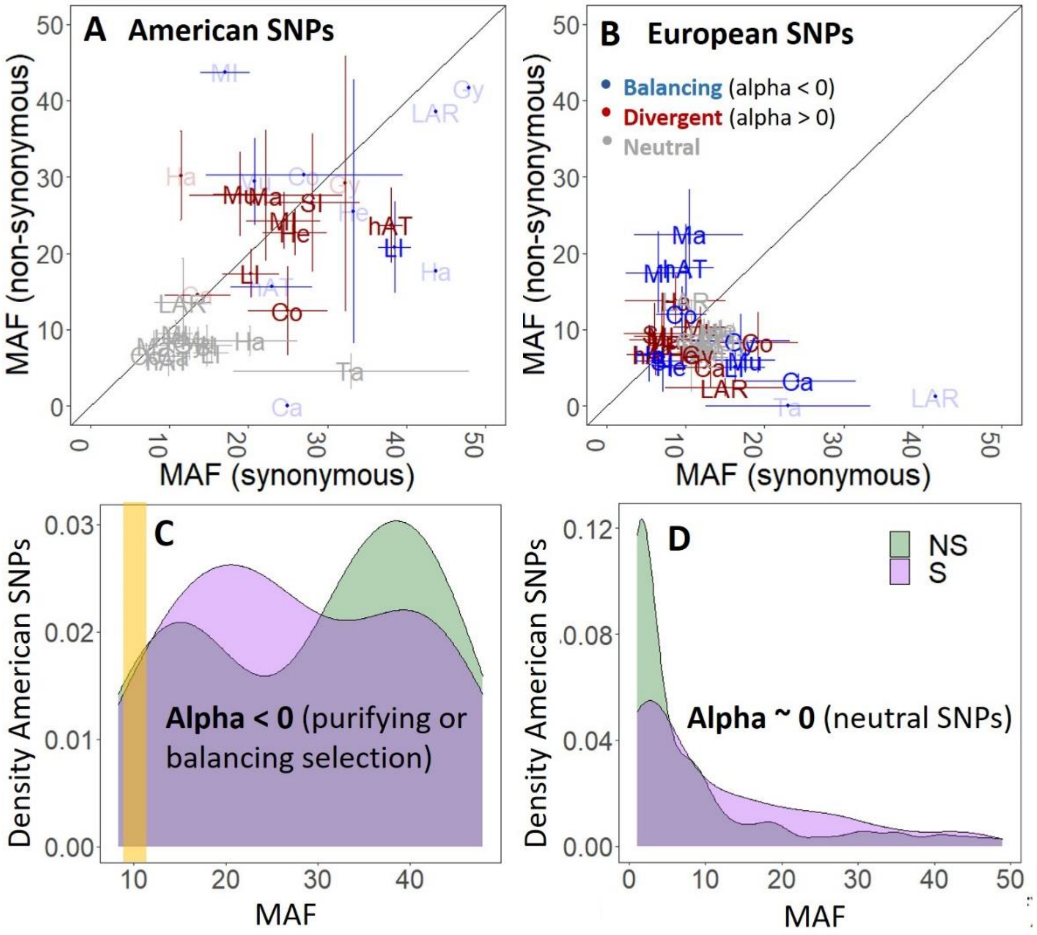

Supplementary Figure 2. **Minimum allele frequency (MAF) distributions.** Panels A and B present non-synonymous vs. synonymous SNPs, for the American (**A**) and European (**B**) *A. lyrata* lineage. MAF means are presented with standard errors per TE superfamily. TE superfamilies with too low sample sizes (N=1) are transparent (e.g. LAR in both panels). TE superfamilies associated with low frequency signatures of balancing selection are considered as candidates of purifying selection if their MAF is smaller than expected under neutrality, i.e. smaller than MAF of neutral SNPs, and smaller for non-synonyomus than for synonymous codon usage. We find that neutral SNPs have the same MAF distribution in both lineages. For the American lineage, we further find that all TE superfamilies with N>1 are characterized by high frequency signatures of selection, indicating little purifying selection. For the European lineage, however, we identify signatures of purifying selection in several TE superfamilies (e.g. MuDR and Cacta), where MAF was smaller for SNPs with signatures of balancing selection than for neutral SNPs, and where non-synonymous SNPs in particular were associated with low MAF. Panels C and D represent MAF density distributions across all non-synonymous (green) and synonymous (purple) SNPs, with signatures of balancing and purifying selection (**C**) vs. neutral SNPs (**D**). The orange rectangle in panel C represents the 95% CI of the mean maf of neutral SNP (9.02-10.64); therefore, all SNPs in panel C left from this orange rectangle are candidates for purifying selection (non-synonymous SNPs with low maf; N=1) and/or for associative overdominance (non-synonymous and synonymous SNPs; N=1 + 0). A total of 37 SNPs (97.4% out of all SNPs with alpha <0) are candidates for real balancing selection arising from heterozygote advantage or frequency-dependent selection.

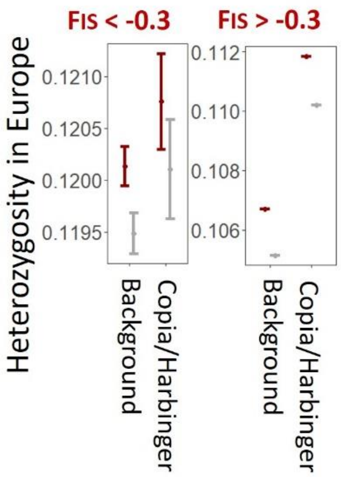

Supplementary Figure 3. Probability of a SNP being heterozygous near Copia and Harbinger elements vs. all other transposable elements. The results (probability estimates obtained by mixed Model 3) are presented separately for *A. lyrata* plants characterized by relatively low (left panel) vs. high (right panel) genome-wide inbreeding coefficients. See Supplementary Data 8 for associated summary statistics. Data are presented as mean values +/- SEM. Figure is based on 4398 SNPs x 4 genetically distinct clusters = 17,592 independent data points.

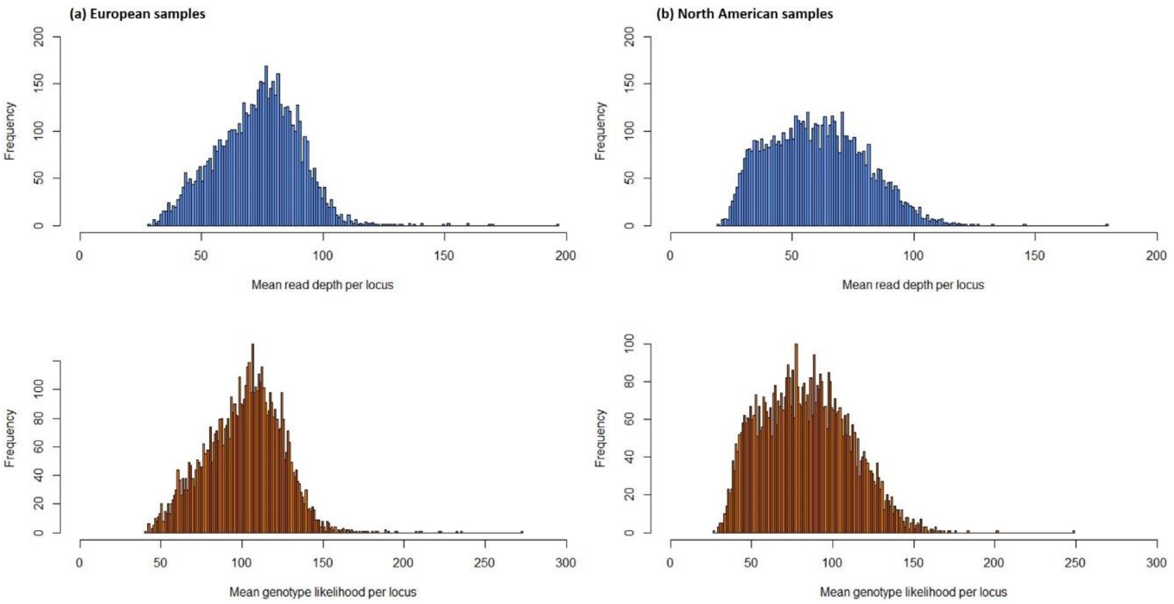

Supplementary Figure 4. Distribution of mean read depth and mean genotype likelihood across samples. (**a**) European samples and (**b**) North American samples for SNPs included in the study.
