# Supplementary material for: Transposable elements maintain genome-wide heterozygosity in inbred populations
Source: Nat Commun. 2022 Nov 17;13:7022. doi: 10.1038/s41467-022-34795-4 (PMC9672359; doi:10.1038/s41467-022-34795-4)
Supplement: Supplementary file 3 — Description of Additional Supplementary Files [file 41467_2022_34795_MOESM3_ESM.pdf]

Description of Additional Supplementary Files

File Name: Supplementary Data 1
Description: American SNPs, their association with their nearest TE, their outlier status (neutral, under divergent selection, under balancing selection), and whether they are homozygous (0) our heterozygous (1) in each individual. Sense represents the orientation of a SNP relative to its nearest TE (downstream vs. upstream of TE vs. within TE), while Sense_Gene represents the orientation of the nearest TE to its nearest gene (downstream vs. upstream of gene, or intragenic). Minor allele frequencies (maf) and substitution type (non-synonymous vs. synonymous) are also provided. Mutation refers to codon usage of SNPs (S = synonymous; NS = non-synonymous; NA = not available). The gene ontology terms (GOs) associated with the SNP reads as obtained from Blast2go are required for GO enrichment analysis.

File Name: Supplementary Data 2
Description: European SNPs, their association with their nearest TE, their outlier status (neutral, under divergent selection, under balancing selection), and whether they are homozygous (0) our heterozygous (1) in each individual. Sense represents the orientation of a SNP relative to its nearest TE (downstream vs. upstream of TE vs. within TE). Mutation refers to codon usage of SNPs (S = synonymous; NS = non-synonymous; NA = not available).

File Name: Supplementary Data 3
Description: American SNPs in VCF Format.

File Name: Supplementary Data 4
Description: European SNPs in VCF format.

File Name: Supplementary Data 5
Description: *Arabisopsis lyrata* samples with their geographical coordinates, predominant mating system (Outcrossing vs. Self-fertilization) and average inbreeding coefficient (Fis). "Cluster" represents the genetic groups as detected by our PCadapt analysis (see Supplementary Data 7 "PCadapt").

File Name: Supplementary Data 6
Description: Output and ANOVA tables of mixed models testing TE effects on nearby heterozygosity. In Model 0, we explored both samples with extreme inbreeding coefficients (Fis > 0.6; self-fertilizing populations) and with reduced inbreeding (Fis < 0.6; outcrossing populations) to assess the effect of TEs on heterozygosity under inbreeding. Model 2 was run on SNPs associated with TEs that significantly affected heterozygosity in Model 0 (cfr. HTE) as well as on a backgorund SNPs (cfr. H0). Model 3 was applied to the European samples to test the hypothesis that H0 = HTE in an outbred dataset (European samples). Distance reflects both a linear and quadratic effect of genomic distance between a SNP and its nearest TE.

File Name: Supplementary Data 7
Description: PCadapt analysis aiming to explore the genetic relatedness among samples (population genetic structure), and to identify SNPs that significantly deviate from this background genetic structure (see Supplementary Data 1 and 2 for SNPs under selection).

File Name: Supplementary Data 8
Description: Gene ontology term enrichment (Chi-square test without correction for multiple testing) for (i) SNPs downstream of TEs, (ii) SNPs upstream of TEs, (iii) divergent outliers, (iv) balancing outliers, (v) SNPs near Copia and Harbinger, (vi) SNPs downstream of Helitrons (vii) SNPs downstream of MuDRs (viii) SNPs downstream of MITEs, and (ix) SNPs downstream of LINEs (x).

File Name: Supplementary Data 9
Description: Two-sided model output related to our third hypothesis (Fig. 1: Q3). First, Pearson correlations are provided for outlier proportions (per TE superfamily) between North American and European data, depending on orientation of SNP relative to TE (downstream vs. upstream). Second, we provide output and ANOVA tables of mixed model 3 testing the TE Effect on nearby heterozygosity in the European dataset. The European dataset was divided into north-European populations characterized by a demographic bottleneck (FIS > -0.3) and central European populations with FIS < -0.3.
