# Supplementary material for: Transposable elements maintain genome-wide heterozygosity in inbred populations
Source: Nat Commun. 2022 Nov 17;13:7022. doi: 10.1038/s41467-022-34795-4 (PMC9672359; doi:10.1038/s41467-022-34795-4)
Supplement: Supplementary file 13 — Reporting Summary [file 41467_2022_34795_MOESM13_ESM.pdf]

nature portfolio

Corresponding author(s): Hanne De Kort

Last updated by author(s): November/01/22

Reporting Summary

Nature Portfolio wishes to improve the reproducibility of the work that we publish. This form provides structure for consistency and transparency in reporting. For further information on Nature Portfolio policies, see our Editorial Policies and the Editorial Policy Checklist.

Statistics

For all statistical analyses, confirm that the following items are present in the figure legend, table legend, main text, or Methods section.

n/a	Confirmed	
☐	☒	The exact sample size (*n*) for each experimental group/condition, given as a discrete number and unit of measurement
☐	☒	A statement on whether measurements were taken from distinct samples or whether the same sample was measured repeatedly
☐	☒	The statistical test(s) used AND whether they are one- or two-sided<br>*Only common tests should be described solely by name; describe more complex techniques in the Methods section.*
☐	☒	A description of all covariates tested
☐	☒	A description of any assumptions or corrections, such as tests of normality and adjustment for multiple comparisons
☐	☒	A full description of the statistical parameters including central tendency (e.g. means) or other basic estimates (e.g. regression coefficient) AND variation (e.g. standard deviation) or associated estimates of uncertainty (e.g. confidence intervals)
☐	☒	For null hypothesis testing, the test statistic (e.g. *F*, *t*, *r*) with confidence intervals, effect sizes, degrees of freedom and *P* value noted<br>*Give P values as exact values whenever suitable.*
☒	☐	For Bayesian analysis, information on the choice of priors and Markov chain Monte Carlo settings
☒	☐	For hierarchical and complex designs, identification of the appropriate level for tests and full reporting of outcomes
☒	☐	Estimates of effect sizes (e.g. Cohen's *d*, Pearson's *r*), indicating how they were calculated

Our web collection on statistics for biologists contains articles on many of the points above.

Software and code

Policy information about availability of computer code

Data collection	SNPs were collected by Buckley et al. 2018 (doi.org/10.1186/s12864-018-4806-7), and TE data were generated by Legrand et al. 2019 (doi.org/10.1186/s13100-019-0171-6). No software was used for data collection.
Data analysis	All data were analyzed using R (version 4.0.3). The R packages include hierfstat 0.5-7, glmmTMB 1.1.3, lme4 1.1-29, TopGo 2.40.0 and pcadapt 4.3.3. Code is published along with the manuscript.

For manuscripts utilizing custom algorithms or software that are central to the research but not yet described in published literature, software must be made available to editors and reviewers. We strongly encourage code deposition in a community repository (e.g. GitHub). See the Nature Portfolio guidelines for submitting code & software for further information.

Data

Policy information about availability of data

All manuscripts must include a data availability statement. This statement should provide the following information, where applicable:

- Accession codes, unique identifiers, or web links for publicly available datasets
- A description of any restrictions on data availability
- For clinical datasets or third party data, please ensure that the statement adheres to our policy

We refer to the NCBI-SRA database BioProject PRJNA495003 for the transposon dataset also used in this study (https://www.ncbi.nlm.nih.gov/bioproject/?term=PRJNA495003). The raw, demultiplexed fastq files can be found on the NCBI SRA database with reference SRP148549 (https://www.ncbi.nlm.nih.gov/sra/?term=SRP148549) . The IDs for the demultiplexed FASTQ files for individual biosamples are SAMN09230090-SAMN09230180 (https://www.ncbi.nlm.nih.gov/bioproject/472246). Source data for figures are provided as zipped text files.

Field-specific reporting

Please select the one below that is the best fit for your research. If you are not sure, read the appropriate sections before making your selection.

☐ Life sciences ☐ Behavioural & social sciences ☒ Ecological, evolutionary & environmental sciences

For a reference copy of the document with all sections, see nature.com/documents/nr-reporting-summary-flat.pdf

Ecological, evolutionary & environmental sciences study design

All studies must disclose on these points even when the disclosure is negative.

Study description	The study combines genome-wide SNP and transposon data in Arabidopsis lyrata. Transposons were annotated in the reference genome, (doi.org/10.1186/s13100-019-0171-6) while SNP data were generated across a sampling range spanning North America and Europe (doi.org/10.1186/s12864-018-4806-7). Heterozygosity and signatures of adaptive evolution were calculated across the genome and analyzed in function of transposon characteristics.
Research sample	A total of 91 individuals of Arabidopsis lyrata were sampled in 13 North American and 18 Northern European populations varying in the degree of inbreeding. A. lyrata is a mixed mating species, making it ideal for studying the role of transposable elements in evolution depending on inbreeding levels. The 91 samples were investigated for SNPs and TE that were collected in previous studies, as described in the "Study description" section of this reporting summary.
Sampling strategy	The samples cover a broad range of genome-wide inbreeding coefficients, which is the key condition upon which our analysis rely. Sample sizes are sufficient for our study since they capture a wide gradient in inbreeding coefficients.
Data collection	Samples for SNP genotyping were collected in Buckley et al. 2018 (doi.org/10.1186/s12864-018-4806-7). Leaves were collected on silica in situ prior to DNA extraction.
Timing and spatial scale	Seeds collected from individual plants were sampled at 12 sites (four selfing, and eight outcrossing populations) across the North American Great Lakes region in the summer of 2011. Samples from European populations were collected from individual plants in the summer of 2007, apart from the leaf tissue from German and Austrian plants, which was collected in 2012. All sampling specifications are provided in the doi's provided in the study description section of this summary.
Data exclusions	One population was excluded because it only contained one sample.
Reproducibility	Because the analyses are based on existing data, they can be repeated based on the codes provided.
Randomization	Random variables were used in the mixed model analyses where relatedness among data points was an issue (e.g. transposons into transposon superfamilies). Using a genetic cluster analysis (R package PCadapt), samples were allocated to genetically similar clusters. These clusters were included as a random factor in our models.
Blinding	Blinding is not applicable to our study because it is based on existing data.

Did the study involve field work? ☐ Yes ☒ No

Reporting for specific materials, systems and methods

We require information from authors about some types of materials, experimental systems and methods used in many studies. Here, indicate whether each material, system or method listed is relevant to your study. If you are not sure if a list item applies to your research, read the appropriate section before selecting a response.

Materials & experimental systems

n/a	Involved in the study
☒	☐ Antibodies
☒	☐ Eukaryotic cell lines
☒	☐ Palaeontology and archaeology
☒	☐ Animals and other organisms
☒	☐ Human research participants
☒	☐ Clinical data
☒	☐ Dual use research of concern

Methods

n/a	Involved in the study
☒	☐ ChIP-seq
☒	☐ Flow cytometry
☒	☐ MRI-based neuroimaging
